# Urokinase Plasminogen Receptor and the Fibrinolytic Complex Play a Role in Nerve Repair after Nerve Crush in Mice, and in Human Neuropathies

**DOI:** 10.1371/journal.pone.0032059

**Published:** 2012-02-21

**Authors:** Cristina Rivellini, Giorgia Dina, Emanuela Porrello, Federica Cerri, Marina Scarlato, Teuta Domi, Daniela Ungaro, Ubaldo Del Carro, Alessandra Bolino, Angelo Quattrini, Giancarlo Comi, Stefano C. Previtali

**Affiliations:** 1 Institute of Experimental Neurology (INSPE), San Raffaele Scientific Institute, Milan, Italy; 2 Division of Neuroscience, San Raffaele Scientific Institute, Milan, Italy; 3 Department of Neurology, San Raffaele Scientific Institute, Milan, Italy; 4 Dulbecco Telethon Institute, Rome, Italy; 5 “Vita e Salute” San Raffaele University, Milan, Italy; Imperial College London, United Kingdom

## Abstract

Remodeling of extracellular matrix (ECM) is a critical step in peripheral nerve regeneration. In fact, in human neuropathies, endoneurial ECM enriched in fibrin and vitronectin associates with poor regeneration and worse clinical prognosis. Accordingly in animal models, modification of the fibrinolytic complex activity has profound effects on nerve regeneration: high fibrinolytic activity and low levels of fibrin correlate with better nerve regeneration. The urokinase plasminogen receptor (uPAR) is a major component of the fibrinolytic complex, and binding to urokinase plasminogen activator (uPA) promotes fibrinolysis and cell movement. uPAR is expressed in peripheral nerves, however, little is known on its potential function on nerve development and regeneration. Thus, we investigated uPAR null mice and observed that uPAR is dispensable for nerve development, whereas, loss of uPAR affects nerve regeneration. uPAR null mice showed reduced nerve repair after sciatic nerve crush. This was a consequence of reduced fibrinolytic activity and increased deposition of endoneurial fibrin and vitronectin. Exogenous fibrinolysis in uPAR null mice rescued nerve repair after sciatic nerve crush. Finally, we measured the fibrinolytic activity in sural nerve biopsies from patients with peripheral neuropathies. We showed that neuropathies with defective regeneration had reduced fibrinolytic activity. On the contrary, neuropathies with signs of active regeneration displayed higher fibrinolytic activity. Overall, our results suggest that enforced fibrinolysis may facilitate regeneration and outcome of peripheral neuropathies.

## Introduction

Nerve regeneration is a critical step in the outcome of peripheral neuropathies. It depends on a combination of signals that control Schwann cell-axon interaction, ensheathment and remyelination. Incomplete regeneration contributes to clinical severity [1].

Extracellular matrix (ECM) components and their receptors play a major role in nerve regeneration [2], [3], [4]. Indeed, we recently reported that endoneurial ECM composition is instructive on nerve regeneration and neuropathy outcome [5]. Nerves enriched with fibrin and vitronectin display insufficient regeneration and poor clinical outcome. Conversely, nerves devoid of fibrin and vitronectin present abundant signs of regeneration and better clinical outcome [5]. Whether the abnormal ECM composition of non-regenerating human nerves is the consequence of impaired fibrinolysis is not known. If fibrinolysis plays an active role for nerve regeneration, modulating fibrinolysis may represent a therapeutic intervention to favor nerve repair.

Studies in rodents showed that fibrinolytic rearrangement of the ECM influences peripheral nerve regeneration. Mice lacking fibrinolytic molecules such as tissue (tPA) and urokinase plasminogen activator (uPA), or plasminogen (plg) show impaired nerve regeneration after damage [6]. Among molecules of the fibrinolytic complex, there is emerging interest in the role of uPA receptor (uPAR), as it is involved either in ECM proteolysis or inflammatory cell migration and adhesion [7], [8]. In peripheral nerves, uPAR is expressed in sensory neurons and Schwann cells [9], and this expression is modulated after nerve damage [10]. However, the role of uPAR in peripheral nerve function and regeneration has never been investigated.

Here we report that uPAR participates in nerve repair and the fibrinolytic complex activity parallels nerve regeneration in human neuropathies.

## Materials and Methods

### Ethics Statement

All the experiments were performed in 2-months-old mice and approved by the Italian regulations and San Raffaele Institutional Animal Care and Use Committee (IACUC 487).

For humans samples, written informed consent was received and accepted from all patients, and the study approved by the San Raffaele Ethical Committee.

### Mice

uPAR−/− mice, previously described [11], were maintained in congenic C57BL/6 strain.

### Neurophysiology

Ten uPAR null mice and 10 control littermates (2 months old) were analyzed for sciatic nerve conduction velocity (NCV) and compound motor action potential (cMAP) in intact nerves, or 45 days post crush as reported [12]. Mice were anesthetized with avertin and placed under a heating lamp to avoid hypothermia. Sciatic NCVs were obtained by stimulating the nerve with steel monopolar needle electrodes. A pair of stimulating electrodes was inserted subcutaneously near the nerve at the ankle. A second pair of electrodes was placed at the sciatic notch, to obtain two distinct sites of stimulation, proximal and distal along the nerve. The muscular response to the electrical nerve stimulation (cMAP) was recorded with a pair of needle electrodes; the active electrode was inserted in muscles in the middle of the paw, while the reference was placed in the skin between the first and second digit.

### Histology and morphometry

Histological analysis of sciatic nerves from mice and sural nerves from human biopsies were performed as described [13]. For mouse morphometry, digitalized images of fiber cross sections were obtained from corresponding levels of the sciatic nerve with a digital camera (Leica DFC300F) using a 100× objective of light microscope (Olympus BX51). At least 5 images from 4 different animals per genotype at each evaluation were acquired (25×10^3^ µm^2^ of sciatic nerve per each animal). The morphometry on semithin sections was analyzed with the Leica QWin software [12]. The ratio between the mean diameter of an axon and the mean diameter of the fiber including myelin (g-ratio), was determined on approximately 200 randomly chosen fibers per animal (3 animals per genotype).

### Immunohistochemistry and Western blotting

These methods have been previously described [12]. Antibodies used for immunohistochemistry/Western blotting included: anti-calnexin and beta-tubulin TUB2.1 (Sigma); collagen IV, fibronectin, Fibrin, Mac1, Neurofilament-160, laminin-beta1, laminin-beta2 (Millipore); Laminin-alpha2 (4H8-2; Alexis); laminin-alpha4 (Santa Cruz); MBP (SMI-94R; Covance); vitronectin (BioTrend).

### Sciatic nerve crush

Adult mice were anesthetized with avertin (trichloroethanol, 0.4 mg/gr of body weight and crush injury performed as described [14]. After skin incision, the sciatic nerve was exposed and crushed distal to the sciatic notch for 20 seconds with fine forceps previously cooled in dry ice. To identify the site of injury, forceps were previously dropped into vital carbon. The nerve was replaced under the muscle and the incision sutured. All the experiments have been performed on the distal portion of the crushed nerve.

### Mouse DRG cocultures

Mouse DRGs and cocultures were established as described [15]. Briefly, mouse DRGs were isolated from E13.5 embryos and placed in C-media containing MEM, 10% fetal calf serum, 2%glucose, 2 mM glutamine, purchased from Life Science and 50 ng/ml Nerve Growth Factor (NGF, from Serotec) on collagen coated glass coverslips. The day after the C-media was removed and replaced for 4–5 days with Neurobasal medium and B27 (vol 1∶1, Life Sciences) without serum and supplemented with NGF, glucose and glutamine as above. Glass coverslips were pre-coated with PLL (final concentration 0.1 mg/ml) and subsequently coated with collagen (rat collagen 0.5 mg/ml from Trevigen). Myelination was induced by treatment for 14 days with C-media supplemented by ascorbic acid (final concentration 50 mg/ml, Sigma).

### Patients and nerve biopsies

Sural nerve samples from 24 patients undergoing nerve biopsy for diagnostic reasons were included in the study. Patient's characteristics are reported in Table 1. Patients were classified as regenerating or non-regenerating on the basis of their histological characteristics at the time of the diagnosis of axonal neuropathy as described [5]. In brief, we considered regenerating nerves those with abundant signs of regeneration (Index of regenerating axons was calculated as the number of regenerating clusters per mm^2^ of sural nerve as follow: 0<40 clusters, 1 = 41–100, 2 = 101–200, 3>201). Severity of the neuropathy was assigned counting large/medium-sized (5–12 µm) myelinated axons in 6–8 representative areas of sural nerve (0.22–0.28 mm^2^); each sural nerve was classified accordingly to the mean number of large/medium-sized myelinated axons per mm^2^ as follow: very severe <200 axons, severe = 201–500, moderate = 501–1400, mild >1401. The neuropathy was diagnosed by the presence of distal sensory disturbances with progressive weakness at four limbs, reduced/absent deep tendon reflexes and neurophysiology showing reduction of motor/sensory nerve action potentials with variable slowing of motor/sensory nerve conduction velocities. Patients underwent routine laboratory tests including screening for disimmune/inflammatory disorders, liver and kidney function, glucose intolerance, muscle enzymes, and cerebrospinal fluid examination. Two independent physicians performed clinical and histopathological evaluations blindly.

**Table 1:** Characterization of human nerve biopsies and evaluation of fibrinolytic activity.

Pt.	Age/Sex	Disease	Severity of the neuropathy	duration	Ax Reg	Fibrinolytic activity	FG	VN	FN
*regenerating patients*									
1*	55/F	polyarteritis nodosa	moderate	36 m	3	2	0.5	0	2
2*	76/F	Wegener granulomatosis	moderate	40 m	4	3	0	0.5	2
3*	62/M	UCTD	mild	24 m	3	2	0	0	2
4*	69/F	HCV mix cryoglobulinemia	moderate	24 m	3	3	0.5	0	3
5	72/F	Chr infl ax neurop	moderate	5 y	2	2	0.5	0.5	2
6*	62/M	Chr infl ax neurop	mild	24 m	3	2	0	0	1
7*	38/M	Chr infl ax neurop	moderate	24 m	3	3	0	0	0.5
8	40/M	CIDP	mild	2 y	1	2	1	1	2
9	48/M	CIDP	mild	3 m	2	3	1	0,5	2
10	44/M	CIDP	moderate	12 m	1	2	1	0,5	2
11*	73/M	idiopathic axonal np	moderate	48 m	2	3	0.5	0	2
12*	66/F	CMT2	mild	24 m	2	2	0	0	1
13	56/F	CMT2	moderate	25 y	1	2	1	0,5	1
*average (+SEM)*					*2.3 (0.06)*	*2.4 (0.05)*	*0.4 (0.05)*	*0.2 (0.05)*	*1.8 (0.05)*
*non regenerating patients*									
14*	60/M	polyarteritis nodosa	severe	48 m	0	0,5	0,5	1	1
15*	58/M	Wegener granulomatosis	very severe	18 m	0	0,5	2	0	3
16*	26/F	Wegener granulomatosis	mild	6 m	0	0,5	2	2	1
17	51/M	Wegener granulomatosis	moderate	4 m	0	1	3	2	1
18*	24M	Chr infl ax neurop	moderate	4 m	0	0,5	3	3	1
19	69/F	CIDP	moderate	3 m	0	0,5	2	2	1
20	30/F	CIDP	very severe	8 y	0	1	3	2	1
21*	65/M	amyloidosis	very severe	24 m	0	1	2	1	1
22*	65/M	idiopathic axonal np	moderate	24 m	0	0,5	1	1	1
23	53/F	idiophatic axonal np	very severe	36M	0	0,5	2	2	1
24*	69/M	toxic neuropathy	severe	3 m	0	0,5	2	1	1
*average (+SEM)*					0	0.6 (0.04)	*2.1 (0.08)*	1.5 (0.08)	1.2 (0.07)
		*t test (reg vs non-reg)*			*p<0.001*	*p<0.001*	*p<0.001*	*p<0.001*	*p = 0.02*

**Legend**: CIDP: chronic inflammatory demyelinating neuropathy; CMT: Charcot-Marie-Tooth neuropathy; UCTD: undifferentiated connective tissue disease; ax: axonal; np: neuropathy. FG: fibrin; VN: vitronectin; FN: fibronectin; m: months; y: years.

*patients already described in ^5^, respectively pt. #3, 6, 7, 8, 11, 12, 14, 17, 19, 20, 39, 41, 27, 30, 43.

### In situ zymography

We determined the activation of the uPA/plasminogen system in frozen sections by *in situ* caseinolytic zymography using the EnzChek® protease green fluorescence assay kit (Invitrogen), following the protocol previously described [16]. This assay is based on the release of fluorescent BODIPY FL®-labeled peptides by plasmin-dependent caseinolysis. Sural nerve sections were incubated with BODIPY-FL® casein solution (10 µg/ml in Tris-HCl; Molecular Probes) for 3 h at 37°C. Caseinolytic activity in nerve sections generated fluorescein peptides that are viewed at confocal microscopy. To test specificity for uPA, sections were pre-incubated with a serine protease inhibitor such as 0.5 µM tPA-STOP (American Diagnostica) or amiloride (0.1 mM; Sigma) at 37°C for 1 h. Semiquantitative analysis of fluorescence staining was measured by ImageJ (NIH) on images of 0.22–0.26 mm^2^ of sural nerve endoneurium viewed using confocal microscope (Leica SP5). For grading, we measured the mean gray value corresponding to the sum of the gray value of all pixels in the selected area divided by the number of pixels, defined as follows: 0 if mean <1, 0.5 if = 2–10, 1 if = 11–20, 2 if = 21–40, 3 if >41.

### SDS-PAGE Zymography

The uPA and tPA activity was evaluated in cell homogenate and conditioned (C- medium, serum free) media from DRG neuron-Schwann cell cocultures grown for 5, 10, 15 and 20 days with (or without) ascorbic acid (50 mg/ml), or sciatic nerve homogenate from 3 Wt and 3 uPAR−/− mice. Conditioned medium from DRG neuron-Schwann cell cocultures was collected after 6 h of incubation and centrifuged at 14,000 rpm for 5 min to remove detached cells and debris. Proteins in the medium were precipitated by the acetone/trichloroacetic acid procedure and were resuspended in lysis buffer (0.1M Tris-HCl pH 8.0, 0.2% Triton-X100). Cell monolayers were washed twice with ice-cold PBS and lysed on the tissue culture dish by addition of ice-cold lysis buffer. Frozen sciatic nerves dissected from uPAR−/− and wild type littermates were pulverized, sonicated in lysis buffer (0,1M Tris-HCl pH 8,0, 1% Triton-X100, 150 mM NaCl), boiled for 5 minutes, and spun at 14,000 rpm for 5 min at room temperature to eliminate insoluble material. Proteins precipitated from media, cells and nerve homogenate were normalized for protein concentration using Bio-Rad Bradford kit. Forty micrograms of Schwann cell/neuron coculture homogenate, or proteins precipitated from medium, or 25 µg of nerve extract were separated on non-reducing SDS/PAGE (10%), after the gels were washed in 2.5% Triton X100 for 45 minutes and subsequently placed in contact with a casein gel containing 2% non-fat dry milk, 0.01M Tris-HCl, 1.25% agarose, and plasminogen (6.25 ng/ml; Roche), at 37°C. After 16 hours, proteolytic activity areas appear as clear bands on casein gel. The polyacrylamyde gel containing nerve homogenate was stained in 0.125% (w/v) coomassie brilliant blue (G-250 Bio-Rad) in 34% methanol, 17% ammonium sulphate, 3,57% phosphoric acid and destained in 1% acetic acid solution as loading control and were analyzed by densitometry. As positive controls, human uPA (0.04 mM, bands 33 and 54 kDa) and rtPA (Actilyse® 0.01 mM, band 65 kDa) were used.

### Statistical analyses

Statistical analyses were evaluated by two tail Student's *t* test.

## Results

### uPAR−/− mice show normal peripheral nerve development and function

**Figure 1:**
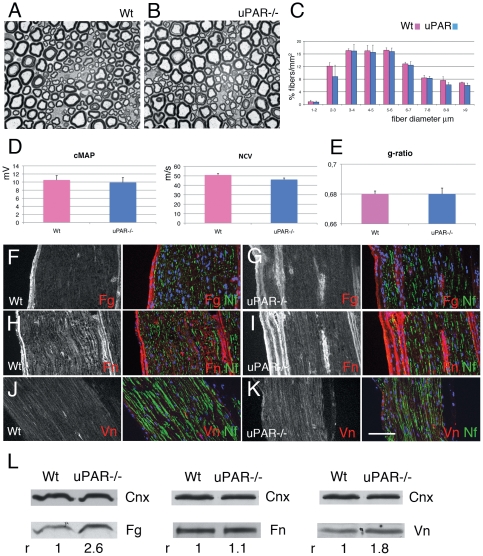
Histological characteristics of uPAR null nerve. (A–B) semithin sections from sciatic nerve of Wt (A) and uPAR null mouse (B) showing normal fiber appearance. (C) Similar fiber type distribution in Wt and uPAR null sciatic nerve. (D) Neurophysiology analysis showing similar values of cMAP and NCV between Wt and uPAR−/− mice (n. 10 mice per group). (E) g-ratio did not show differences in myelin thickness between Wt and uPAR−/− nerves (n. 20000). (F–K) sciatic nerve cryosections of Wt and uPAR null mouse stained for fibrin fibronectin and vitronectin. Fibrin and vitronectin staining was mildly increased in uPAR null endoneurium (G and K) as compared to Wt (F and J), whereas fibronectin was similarly expressed (H–I). (L) Western blot analysis of fibrin and fibronectin in Wt and uPAR null sciatic nerve homogenate. Calnexin was used to normalize loading (fibronectin and vitronectin were loaded on the same gel, hence they have the same calnexin bands). r = densitometric ratio between the band of interest and calnexin; Wt was always assigned as r = 1. Fibrin and vitronectin levels were increased in uPAR null nerves as compared to Wt, whereas levels of fibronectin were similar. Fg = fibrin; Fn = fibronectin; Vn = vitronectin; Nf = neurofilaments. Bar = 15 µm in A and B; 50 µm in F–K.

We assessed whether uPAR loss affects nerve function and structure. Neurophysiological analysis of uPAR−/− mice showed normal nerve conduction velocities and compound motor action potentials (Figure 1D). Consistently, morphological analysis of sciatic nerves did not reveal differences in total fiber number (Wt 18452±270 fibers/mm^2^ vs uPAR−/− 17750±679 fibers/mm^2^), fiber size distribution and myelination (Figure 1A–C and 1E).

As uPAR-uPA complex plays a role in ECM remodeling, we then investigated the endoneurial ECM composition. By immunohistochemistry we did not observe differences in the expression of laminins (α2, β1, β2, γ1), fibronectin, and collagen IV (data not shown). Instead, we observed increased deposition of fibrin and vitronectin in uPAR−/− mice (Figure 1F–K, and not shown). Western blot analysis confirmed increased amount of fibrin and vitronectin in uPAR−/− mice (Figure 1L). Thus, uPAR is dispensable for nerve development.

**Figure 2:**
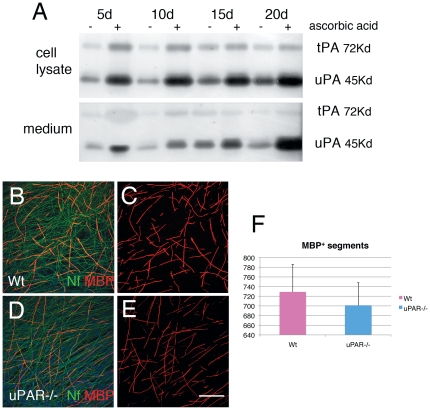
Fibrinolytic molecules in myelination. (A) Time course zymography of uPA and tPA activity in Wt DRG explant homogenate (as a pool of at least 8 coverslips) and their conditioned media without ascorbic acid treatment (-) or after 5, 10, 15, 20 days of ascorbic acid (+). Note both tPA and uPA activity are induced after ascorbic acid in both cell homogenate and media, although uPA activity increases in parallel with myelination. (B–E) DRG explants from Wt and uPAR null mice stained for neurofilament (green) and MBP (red) 7 days after ascorbic acid. The number of myelinated segments were similar between Wt and uPAR null explants. Bar = 50 µm.

Since *in vitro* system may unravel defects masked by compensatory or redundant molecules during development *in vivo*, we exploited uPAR−/− Schwann cell-DRG neuron cocultures. As a preliminary experiment, we evaluated whether fibrinolytic activity is present and modulated in cocultures. Thus we evaluated tPA and uPA activity by zymography in organotypic DRG explants from wild type mice, in which myelination was induced by ascorbic acid. We found that both tPA and uPA activity are induced when myelination starts, and that uPA activity increased proportionally with myelination (Figure 2A). We then evaluated myelination of uPAR−/− DRG explants, measured as number of MBP positive segments. Myelination was not significantly different between uPAR−/− and Wt DRG explants seven days after ascorbic acid treatment (Figure 2B–F).

Overall, although uPA and tPA activity parallels myelination, suggesting a role in this process, uPAR is dispensable also *in vitro* for myelination during development.

### uPAR−/− mice present significant reduction of repair after damage

As abnormal fibrin and vitronectin deposition affects nerve regeneration, we evaluated the response to nerve injury in uPAR−/− mice.

**Figure 3:**
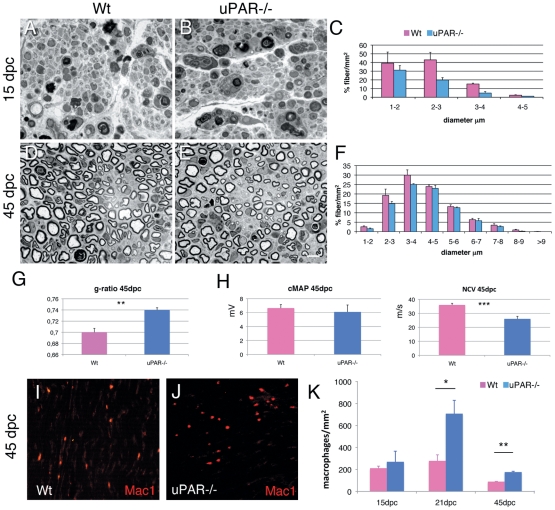
Sciatic nerve regeneration after injury. (A–B, D–E) semithin section and (C, F) fiber type distribution from sciatic nerve of Wt and uPAR null mice at 15 and 45 dpc. At both 15 and 45 dpc we observed reduced number of regenerating fibers. (G) g-ratio was significantly increased in uPAR−/− regenerating fibers at 45 dpc (n. 20000; p = 0.01). (H) Neurophysiological analysis showing similar values of cMAP between Wt and uPAR−/− mice, whereas NCV was significantly reduced in uPAR−/− mice (n. 8; p = 0.001). (I–J) Staining for Mac-1/CD11b (Mac1) in Wt (I) and uPAR null (J) sciatic nerve 45dpc. (K) Quantification of number of macrophages observed in sciatic nerve 15, 21 and 45dpc; differences were significant at 21 and 45 dpc (*p = 0.04; **p = 0.008). Bar = 10 µm in A, B, D, E, I and J.

We performed sciatic crush injury in 12 uPAR−/− and 12 littermate control mice and analyzed peripheral nerve regeneration in the nerve stump distally to the site of the lesion at 15 and 45 days post crush (dpc). Fifteen dpc, both wild type and uPAR−/− nerves showed axonal degeneration, invading macrophages and few regenerating fibers. The number of regenerating fibers was significantly reduced (34%) in uPAR−/− nerves (Wt 5850±430/mm^2^ vs uPAR−/− 3900±250/mm^2^; p = 0.02), and consistent for all fiber diameters (Figure 3A–C). Forty-five dpc, when Wt nerves are usually regenerated, uPAR−/− nerves still showed a significant reduced number (22%) of regenerating fibers (Wt 17800±889 fibers/mm^2^ versus uPAR−/− 14007±260 fibers/mm^2^; p = 0.05); this was primarily evident for small diameter fibers (Figure 3D–F). Consistent with defective regeneration, we also observed that regenerating fibers in uPAR−/− mice had reduced myelin thickness, as measured by g-ratio (Figure 3G; n. 26, p = 0.01). Finally, neurophysiological analysis confirmed impaired regeneration as uPAR−/− mice displayed a significant reduction in NCV as compared to Wt mice at 45 dpc (Figure 3H; n. 8, p = 0.001).

Overall our results suggest that nerve repair is impaired in uPAR−/− mice. As uPAR has been associated to leukocyte migration [7], [17], especially into the nervous system during experimental autoimmune encephalomyelitis [18], we evaluated if impaired nerve repair in uPAR−/− mice was due to reduced macrophage recruitment in the nerve. In fact, macrophage activity is crucial for myelin clearance in damaged nerve to promote efficient nerve repair [1]. However, staining for macrophages using anti-Mac1 antibody did not show a reduced number of macrophages in uPAR−/− nerves (Figure 3I–J). The number of macrophages was similar at 15dpc, while significantly increased in uPAR−/− mice at 21dpc (p = 0.04) and 45dpc (p = 0.008; Figure 3I–K). An increased number of macrophages in uPAR−/− nerve would suggest a delay in clearance of nerve debris, thus in accordance with a delay in nerve regeneration.

### uPAR−/− mice have reduced fibrinolysis

**Figure 4:**
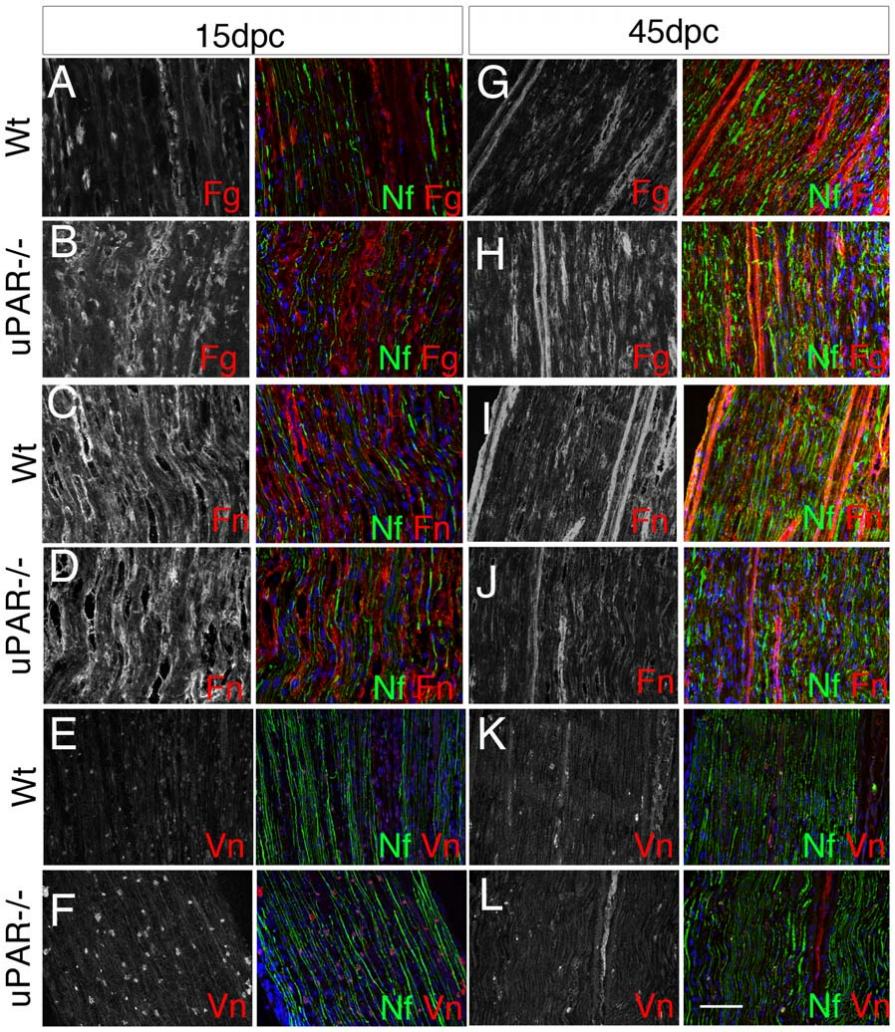
Different expression of ECM in uPAR−/− nerves after damage. Sciatic nerve cryosections from Wt (A, C, E, G, I, K) and uPAR−/− (B, D, F, H, J, L) mice stained for fibrin (Fg), fibronectin (Fn) or vitronectin (Vn), and neurofilaments (Nf) at 15 and 45 dpc. Both at 15 and 45 dpc Fibrin expressions was higher in uPAR−/− as compared to Wt endoneurium (B versus A, and H versus G). Fibronectin expression was similarly in Wt and uPAR−/− mice at both time points (D versus C, and J versus I). Vitronectin expression was higher in uPAR−/− mice as compared to wt at 15dpc (F versus E), whereas it was similar at 45 dpc (L and K). Bar = 50 µm.

**Figure 5:**
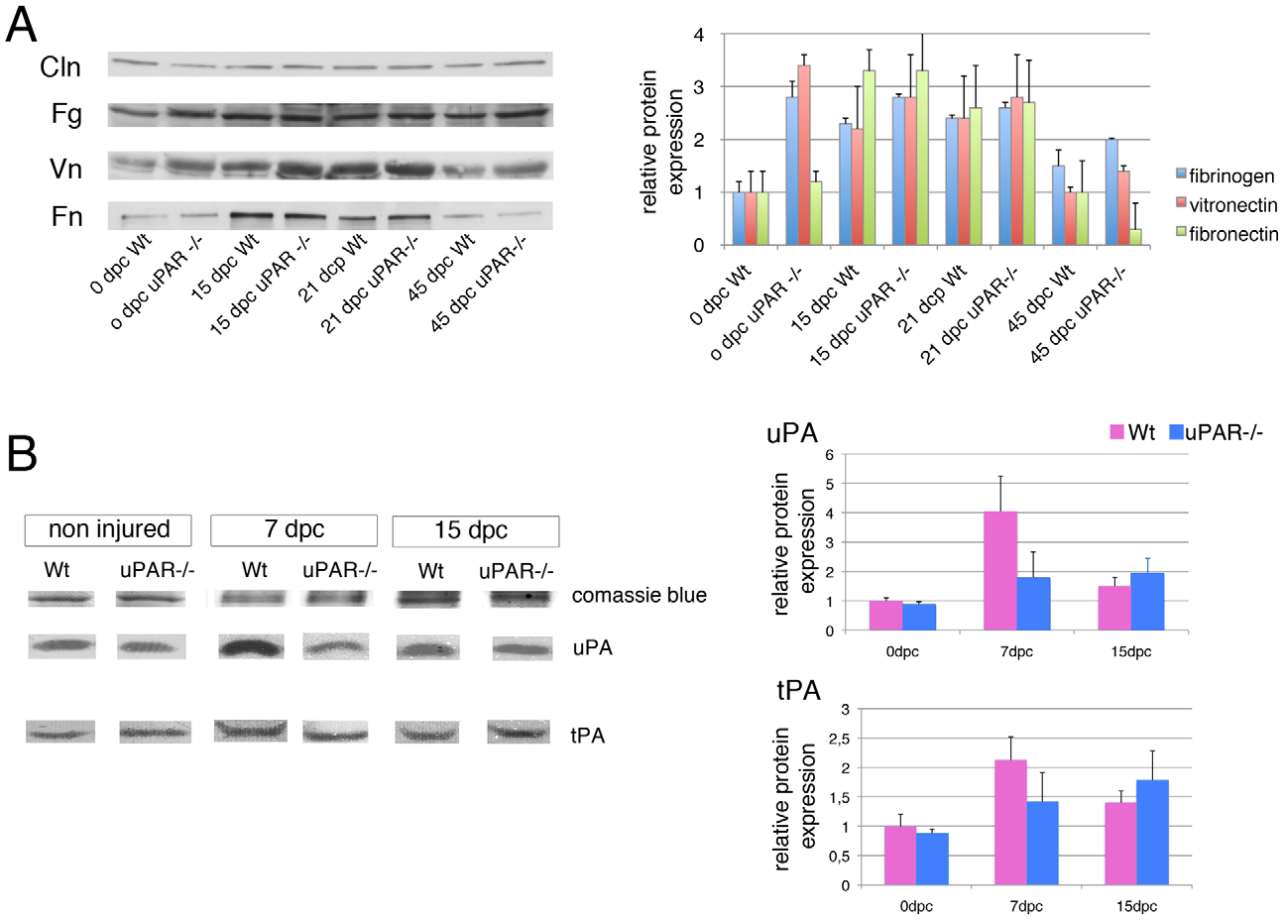
Impaired uPA activity and fibrin clearance in uPAR−/− nerves after damage. (A) western blot analysis of fibrin and fibronectin in Wt and uPAR null sciatic nerve homogenate at 15, 21 and 45 dpc. Calnexin was used to normalize loading. Quantification of western blot is reported as an average of 3 independent experiments, and represented as ratio fibrin/calnexin, vitronectin/calnexin and fibronectin/calnexin, assigning Wt 0dpc as 1±SEM). At each time point uPAR null homogenate showed increased levels of fibrin and vitronectin as compared to Wt, whereas fibronectin levels were higher at 15 and 21 dpc, but lower at 45 dpc. (B) Zymography of sciatic nerve homogenate from Wt and uPAR null mice measuring tPA and uPA activity 0, 7 and 15dpc. Bands were stained with Coomassie blue as loading control. Quantification of zymography is reported as an average of 3 independent experiments, and represented as ratio uPA/coomassie blue and tPA/coomassie blue, assigning Wt 0dpc as 1±SEM. Note the reduced increase of uPA in uPAR null homogenate as compared to Wt 7 and 15dpc, whereas there are no differences in the tPA activity between mutant and Wt mice. Fg = fibrin; Fn = fibronectin; Vn = vitronectin; Cln = calnexin.

We then assessed whether defect in nerve repair was mediated by abnormal ECM composition. We focused on fibrin, fibronectin and vitronectin, which are targets of the fibrinolytic complex. As expected, we observed increased expression of fibrin, vitronectin and fibronectin after nerve damage in the endoneurium of both Wt and uPAR−/− mice, although this expression was consistently higher in uPAR−/− mice (Figure 4A–F and Figure 5A; values are expressed as average of 3 experiments±SEM). At 45 dpc, when nerve should be almost recovered, Wt nerves showed roughly normal levels of all three ECM molecules, whereas fibrin and vitronectin levels were still increased in mutant nerves (Figure 4G–L and Figure 5A), and fibronectin levels markedly decreased (Figure 5A). Overall these results suggest that normal remodeling of ECM after damage is impaired in uPAR−/− mice, which consistently have higher levels of fibrin and vitronectin.

We therefore evaluated whether uPAR−/− mice have a defective function of the fibrinolytic complex. Nerve tissue from the crush site of 3 Wt and 3 uPAR−/− mice was obtained at 7 and 15 dpc and from non-injured nerves. tPA and uPA activity was determined by zymography (the experiment was performed 3 times). In non-injured nerve, uPA and tPA activity was similar between uPAR−/− and Wt mice. After nerve damage, uPA and tPA activity was modulated in Wt nerves, reaching a peak at 7dpc and decreasing at 15dpc (Figure 5B). In uPAR−/− mice instead, uPA activity was only mildly increased and not modulated (Figure 5B).

These results demonstrate that uPAR−/− mice have a reduced uPA activity after nerve damage, which sustains their inability to degrade fibrin.

### Exogenous fibrinolytic treatment ameliorate nerve repair in uPAR−/− mice

**Figure 6:**
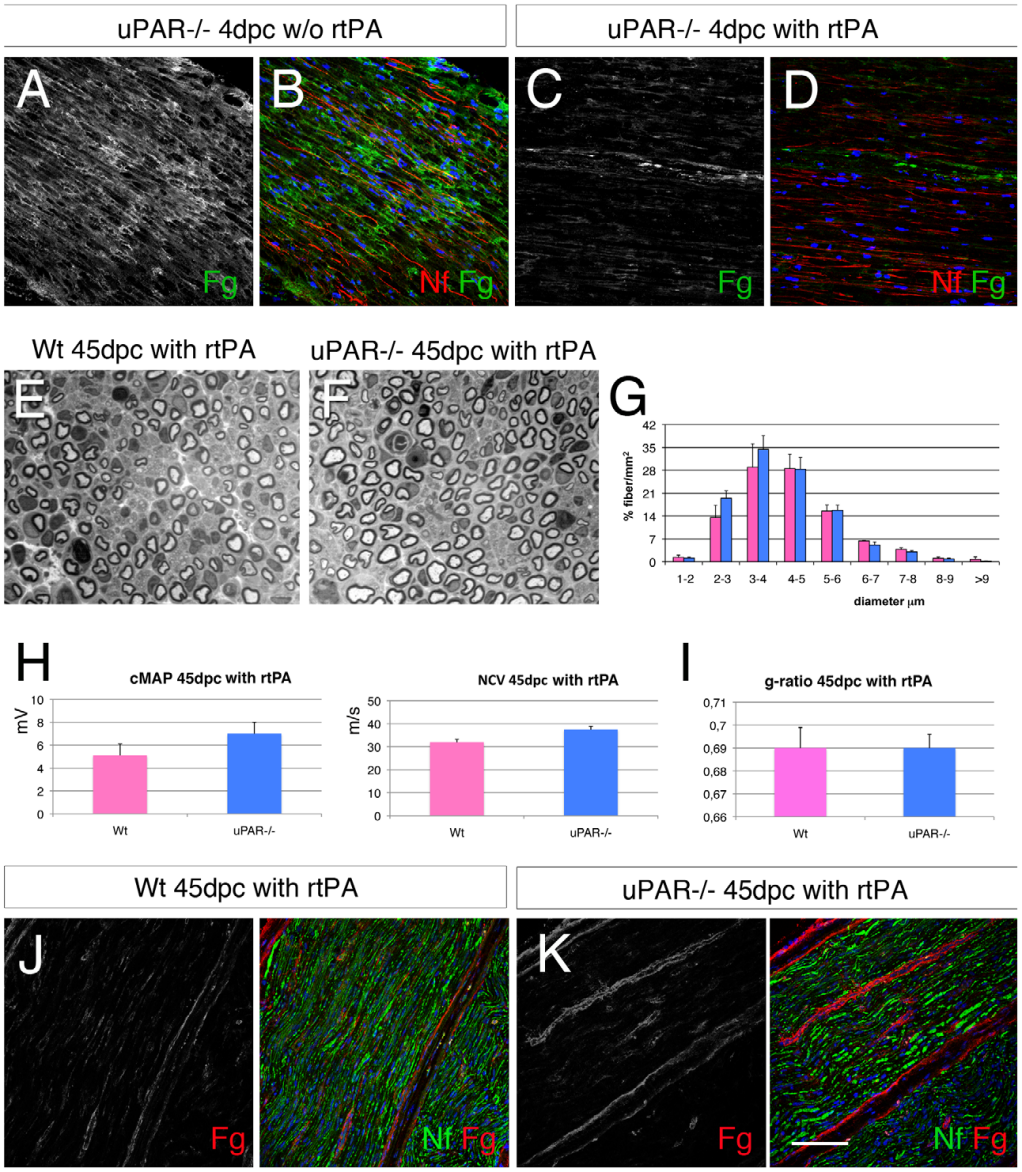
Exogenous recombinant tPA rescues abnormal myelination in uPAR null nerves after injury. (A–D) Sciatic nerve cryosections from Wt and uPAR null mice stained for fibrin 4 dpc without or after exogenous treatment with recombinant tPA (5 mg/100 g). After treatment fibrin staining is mostly abolished (compare C and A). (E–F) semithin sections from sciatic nerve 45 dpc of Wt and uPAR null mice after treatment with recombinant tPA (5 mg/100 g , 2 times per week for 3 weeks), and (G) fiber type distribution. Number and fiber type distribution was similar in the two groups. (H) Neurophysiology analysis showing similar values of cMAP and NCV between Wt and uPAR−/− mice treated with rtPA at 45 dpc. (I) g-ratio did not show differences in myelin thickness between Wt and uPAR−/− nerves (n. 15000). (J–K) immunofluorescence staining for fibrin in sciatic nerves at 45 dpc from mice treated with rtPA, showing low expression of fibrin. Fg = fibrin; Nf = neurofilaments. Bar = 50 µm in A–D and J–K; 20 µm in E–F.

To verify whether reduced fibrin deposition would improve nerve repair in uPAR−/− mice, we performed exogenous fibrinolysis by treating mice with recombinant (r)tPA (5 mg/ml; Actilyse®). We first verified that intra-peritoneal administration of rtPA (roughly 1 mg per mouse) was sufficient to remove fibrin in the endoneurium of uPAR−/− crushed sciatic nerves. Five uPAR−/− and 5 Wt mice have been treated with rtPA either other day from 1dpc and the sciatic nerve analyzed at 4dpc. Fibrin that is highly expressed in the endoneurium of non-treated mice was reduced in the endoneurium of rtPA treated mice (Figure 6A–D).

We then performed sciatic crush in 3 uPAR−/− and 3 Wt mice and treated these mice for 3 weeks, 2 times per week from 1dpc, with rtPA (5 mg/100 g). Forty-five dpc, motor nerve conduction velocities and cMAPs were similar in uPAR null mice and controls (Figure 6H). Accordingly, both wild type and uPAR−/− nerves showed quite complete nerve recovery, and we did not observe differences in total number of fibers (Wt 16300±1082 fibers/mm^2^ vs uPAR−/− 17680±1090 fibers/mm^2^), fiber type distribution (Figure 6E–G) and myelin thickness (Figure 6I). Immunohistochemistry for fibrin expression showed similar pattern in Wt and uPAR−/− mice (Figure 6J–K).

### Human neuropathies show different fibrinolytic complex activity

**Figure 7:**
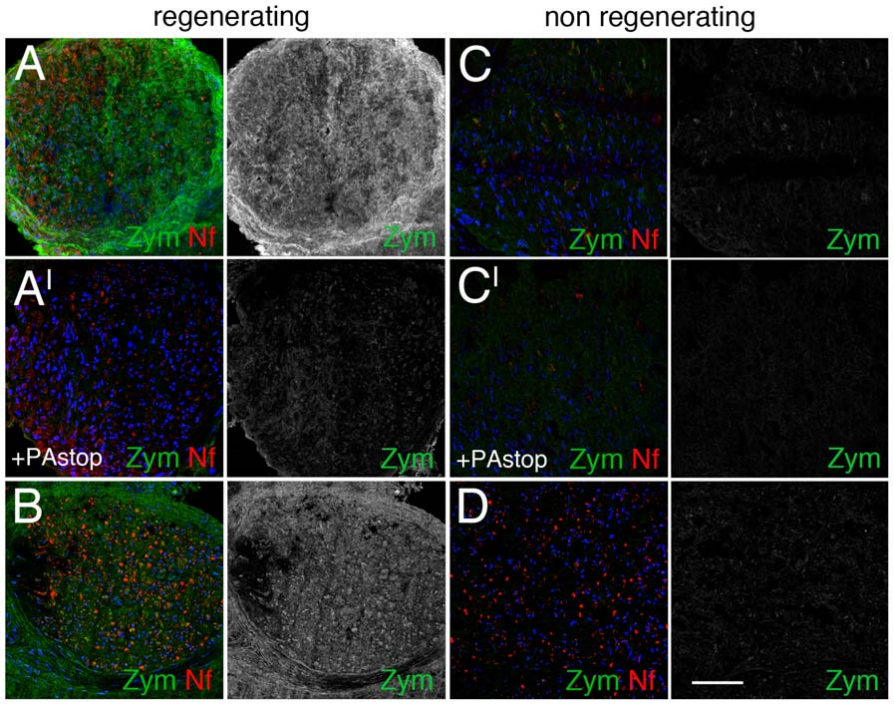
Fibrinolytic activity in human neuropathies. In situ zymography in sural nerve biopsies of patients (#4 and #3) with regenerating (A, B) and (#15 and #18) non-regenerating (C, D) neuropathies. Fluorescent signal as a readout of fibrinolytic activity is very high in regenerating nerves, and very low in non-regenerating nerves. A^I^ and C^I^ are the same reaction depicted in A and C in which fibrinolytic reaction was blocked by amiloride (PAstop). Zym = fibrinolytic activity; Nf = neurofilaments. Bar = 50 µm.

Our previous findings indicate that nerve regeneration in human neuropathies is impaired in the presence of abnormal accumulation of fibrin and vitronectin in the endoneurium [5]. We investigated whether the fibrinolytic activity is involved in the abnormal deposition of fibrin and vitronectin in peripheral nerves of human neuropathies (Table 1). We analyzed 24 nerve biopsies from patients affected by peripheral neuropathy that, on histopathology evaluation, were divided in regenerative (patients #1–13) and non-regenerative (patients #14–24) neuropathies. On these nerve samples, we performed in situ zymography and observed that all the nerves showing higher fibrinolytic activity belong to the group of regenerating neuropathies (Table 1 and Figure 7). This result was independent from the diagnosis of the neuropathy. When an inhibitor of tPA or uPA activity was added before zymography, the fluorescent activity was reduced or abolished, confirming the specificity of the result Figure 7A^I^–B^I^. Consistently, nerves with signs of regeneration and high fibrinolytic activity showed significantly lower expression of fibrin and vitronectin (Table 1). On the contrary, patients with no signs of regeneration and low fibrinolytic activity showed significantly higher expression of fibrin and vitronectin (Table 1).

Overall these data suggest that efficient fibrinolytic activity remodels the endoneurial ECM in order to favor nerve regeneration.

## Discussion

Given the role of the fibrinolytic complex in nerve regeneration, the molecular characterization of its major components is extremely important as they may constitute future targets for therapeutical intervention. We therefore investigated the role of uPAR by loss of function experiments, as its spatial and temporal expression suggested its involvement in nerve function [9], [10]. We observed that uPAR plays a role in nerve regeneration, as it is necessary for proper remodeling of the endoneurial ECM.

Proper fibrin remodeling is critical for nerve repair. Soon after nerve damage, an immature ECM composed by fibrin clot is instrumental for tissue reorganization and successful regeneration [5], [19]. However, to proceed towards regeneration, the fibrin clot has to be removed to form “mature” ECM enriched in fibronectin [5], [20]. Mice devoid of fibrin show accelerated nerve regeneration after injury [19], [21], suggesting that fibrin is a negative regulator of nerve repair. Consistently, in uPAR−/− mice we observed the inverse result. Loss of uPAR causes excessive fibrin and vitronectin deposition due to impaired uPA activity, which results in reduced nerve repair. Consistently, when we forced fibrinolysis by exogenous administration, we reduced endoneurial fibrin deposition and ameliorated nerve repair in uPAR−/− mice. Hence, our results suggest that uPAR is necessary for proper uPA fibrinolytic activity and repair in peripheral nerve.

Leukocytes, including macrophages, express uPAR [7], which mediate adhesion and migration to sites of inflammation through interaction with ECM molecules, mainly vitronectin [17]. In uPAR−/− mice, this lack of interaction is responsible for delayed and less acute spinal cord inflammation in experimental allergic encephalomyelitis [18]. In the peripheral nerve, macrophage recruitment and infiltration at site of nerve injury is fundamental for the removal of myelin and axonal debris, and to promote efficient regeneration. However, conversely to what is described in the central nervous system, loss of uPAR does not reduce macrophage infiltration in the injured nerves. A different repertoire of adhesive receptors is likely involved in the macrophage recruitment into the peripheral nerve, which is alternative to uPAR-vitronectin [22], [23].

This article and others [6], [10], [19], [21], [24], [25] clearly show that the fibrinolytic complex is involved in nerve regeneration in animal models. We already described that remodeling of ECM, and particularly the clearance of fibrin in the endoneurium, is fundamental for nerve regeneration in human neuropathies [5]. Now we show for the first time that the activity of the fibrinolytic complex is different in nerve biopsies of human neuropathies, being higher when the amount of endoneurial fibrin and vitronectin is low, and vice-versa. We showed that neuropathies with defective regeneration, and worse clinical outcome, had reduced fibrinolytic activity. On the contrary, neuropathies with active signs of regeneration, and better clinical outcome, displayed high fibrinolytic activity. These results are in agreement with the idea that enhanced fibrinolysis may ameliorate axonal regrowth after damage and facilitate Schwann cell-axon interaction. Interestingly, our results were independent from the pathogenetic mechanism of the neuropathy, suggesting that this is a general mechanism associated to nerve regeneration. We are tempting to speculate that exogenous fibrinolysis might constitute an associative therapy to ameliorate pathological and clinical outcome of a subset of peripheral neuropathies. On the other hand, drug (Actilyse ®) and dosage we used in this study would not be sufficiently safe to be translated in humans. The high risk for haemorrhages as side effect, makes Actilyse inappropriate for not life threatening disorders such as peripheral neuropathy. Moreover, although in our small case series we did not observe haemorrages in treated mice, careful autopsy was not performed. This important side effect should be investigated in a larger series of mice before envisage any translation in human to treat neuropathies.

In conclusion, our results propose uPAR as a molecule involved in the fibrinolityc activity in peripheral nerve repair. Moreover, our findings sustain that the fibrinolytic complex plays a role in nerve repair in human neuropathies, suggesting that enhanced fibrinolysis may ameliorate the pathology and the outcome of peripheral neuropathies. Further studies with drugs with minor side effects and in selected animal models of human neuropathies would be necessary to address this hypothesis.
